# Homozygosity disequilibrium and its gene regulation

**DOI:** 10.1186/s12919-016-0023-z

**Published:** 2016-10-18

**Authors:** Hsin-Chou Yang, Yu-Ting Lin

**Affiliations:** Institute of Statistical Science, Academia Sinica, Nankang 115, Taipei, Taiwan

## Abstract

Homozygosity disequilibrium (HD) describes a nonrandom pattern of sizable runs of homozygosity (ROH) that deviated from a random distribution of homozygotes and heterozygotes in the genome. In this study, we developed a double-weight local polynomial model for estimating homozygosity intensity. This new estimation method enables considering the local property and genetic information of homozygosity in the human genome when detecting regions of HD. By using this new method, we estimated whole-genome homozygosity intensities by analyzing real whole-genome sequencing data of 959 related individuals from 20 large pedigrees provided by Genetic Analysis Workshop 19 (GAW19). Through the analysis, we derived the distribution of HD in the human genome and provided evidence for the genetic component of natural variation in HD. Generalized estimating equation analysis for 855 related individuals was performed to identify regions of HD associated with diastolic blood pressure (DBP), systolic blood pressure, and hypertension (HTN), with concomitant adjustment for age and sex. We identified one DBP-associated and 2 HTN-associated regions of HD. We also studied the gene regulation of HD by analyzing the real whole-genome transcription data of 647 individuals. A set of gene expressions regulated by the DBP- and HTN-associated regions of HD was identified. Finally, we conducted simulation studies to evaluate the performance of our homozygosity association test. The results showed that the association test had a high power and that type 1 error was controlled. The methods have been integrated into our developed Loss-of-Heterozygosity Analysis Suite software, which can be downloaded at http://www.stat.sinica.edu.tw/hsinchou/genetics/loh/LOHAS.htm.

## Background

Homozygosity disequilibrium (HD) is defined as a nonrandom pattern of sizable runs of homozygosity (ROH) that deviated from a random distribution of homozygotes and heterozygotes in the genome [1]. The 2 types of ROH are: narrow-sense and broad-sense ROHs. A narrow-sense ROH indicates a contiguous stretch of homozygotes in an intact genomic region [2, 3]. A broad-sense ROH entails a homozygosity-rich region that contains a small proportion of heterozygotes, which are caused by genotyping errors, missing genotypes, or mutations [1]. The genetic mechanisms of HD include autozygosity [2], natural selection [4], and chromosomal aberrations [5]. Genetic contributions of HD to the susceptibility of Mendelian diseases, complex disorders, and cancers were reported [6]. A population difference in HD was also reported [7].

We developed statistical methods and software (Loss-of-Heterozygosity Analysis Suite [LOHAS]) to dissect the whole-genome patterns of HD through whole-genome single nucleotide polymorphism (SNP) analysis [7]. Our method adopted a broad-sense definition of ROH. The methods were applied to investigate the association between HD and disease susceptibility [1, 7, 8] and the relationship between HD and the genetic structures of global populations [7]. Because no studies had investigated HD by using whole-genome sequencing (WGS) data, we expanded our methods and software to analyze the WGS data set provided by Genetic Analysis Workshop 18 [9]. The method was based on the assumption that all rare variants (RVs) have an equal weight, even though common homozygotes of RVs with a lower minor allele frequency (MAF) carry less homozygosity information [9].

In this study, we analyzed real WGS and whole-genome transcription (WGT) data and analyzed simulated data from Genetic Analysis Workshop 19 (GAW19) and our previous study [9]. This study aimed to (a) develop new statistical methods and analysis tools for examining HD in WGS data; (b) characterize patterns of HD in the human genome by using WGS data; (c) identify regions of HD associated with diastolic blood pressure (DBP), systolic blood pressure (SBP), and hypertension (HTN); (d) examine gene expression regulated by HD; and (e) evaluate the power and type 1 error of the proposed genome-wide homozygosity association analysis according to simulated data sets.

## Methods

### Materials

GAW19 provided a combined imputation WGS data set, including deep sequencing data for the whole genomes of 464 individuals and genome-wide SNP genotyping data for 495 individuals, derived from 20 large independent pedigrees enrolled in the Type 2 Diabetes Genetic Exploration by Next-generation sequencing in Ethnic Samples (T2D-GENES) Project 2 (filename: chrN-geno.csv.gz). Constituting the 8,348,663 single nucleotide variants (SNVs) were 2,769,837 SNPs and 5,578,826 RVs for odd-numbered autosomes. The clinical data (DBP and SBP), covariates (age and sex), and antihypertensive medication of 855 individuals were available (filename: PHEN.csv). Patients were considered hypertensive if they had ever taken antihypertensive medication or their DBP exceeded 90 mm Hg or SBP exceeded 140 mm Hg at the most recent examination. Among patients who received hypertensive medication, the values of DBP and SBP were increased by 5 mm Hg and 10 mm Hg, respectively, to adjust for the effects of the medication [10]. The WGT data of 647 individuals were generated from peripheral blood mononuclear cells by using Illumina Sentrix Human Whole Genome microarrays (filename: EXPR.csv) [11]. After data preprocessing, there were 20,634 transcripts. In addition, GAW19 provided 200 simulation data sets of quantitative trait Q_1_ from 849 individuals (filename: SIMPHEN.I.csv). Q_1_ was generated from a normal distribution and was independent of genetic variants in this study. Q_1_ was used to evaluate the type 1 error of our homozygosity association test. To assess statistical power, we reanalyzed 200 additional simulation data sets of Q_1_-associated regions of HD generated in our previous study [9]. We selected 3 regions that represented the 10th, 50th and 90th percentiles of proportions of RVs on chromosome 21. Let *p* denote the probability that all genotypes in the region were replaced by homozygotes. The 3 regions were created as Q_1_-associated regions of HD by using the following logistic regression model:

$$ Logit(p)={b}_0+{b}_1\cdot {Q}_1, $$

where *b*_0_ ranged from −25 to −10 with an increment of 5, and *b*_1_ ranged from 0.3 to 1 with an increment of 0.1.

### Statistical methods

We estimated homozygosity intensities on a chromosome as follows. First, we constructed sliding windows on a chromosome by using the nearest neighbor method with a bandwidth of *h*(*b*), which corresponded to the *b*% of SNVs on a chromosome that were contained in each window (in this study, *b*% = 5 %). Second, for each individual and for each window, we estimated homozygosity intensity by using a double-weight local polynomial model. Let {*x*_*i*_, *i* = 1, ⋯, *m*_*c*_} denote the physical position of the *i* th SNV, where *m*_*c*_ denotes the number of SNVs on chromosome *c*. Let {*Y*_*i*_, *i* = 1, ⋯, *m*_*c*_} denote an indicator of the homozygous status of the *i* th SNV, taking value 1 (0) for a homozygous (heterozygous) SNV. The estimator of homozygosity intensity at the physical position of $x\;\left[\mathrm{i}\mathrm{e},\widehat{\lambda}(x)={\widehat{\alpha}}_0\right]$ can be derived by minimizing the locally weighted least squares criterion *E* as follows:

1 $$ E(x)={\displaystyle {\sum}_{i=1}^{m_c}K\left(\frac{x_i-x}{h(b)}\right)}L\left({x}_i\right){\left\{{Y}_i-\left[{\alpha}_0+{\alpha}_1\left({x}_i-x\right)+\cdots +{\alpha}_p{\left({x}_i-x\right)}^p\right]\right\}}^2 $$

In Eq. (1), kernel weight *K*(*u*) was designed to consider a local correlation of SNVs. A higher weight was assigned to an SNV closer to the physical position of *x*. Locus weight *L*(*x*_*i*_) was designed to reduce the weights of the common homozygotes of RVs when defining ROH, because common homozygotes of RVs that had a lower MAF carried less homozygosity information. In this study, we considered cubic kernel weight and locus weight as follows:

$$ K(u)=\left\{\begin{array}{c}\hfill {\left(1-{\left|u\right|}^3\right)}^3,\left|u\right|<1\hfill \\ {}\hfill 0,\kern0.5em \mathrm{otherwise}\hfill \end{array}\kern0.5em \mathrm{and}\kern0.5em \right.L\left({x}_i\right)=\left\{\begin{array}{c}\hfill 1,MA{F}_i\ge 0.05\hfill \\ {}\hfill MA{F}_i/0.05,0\le MA{F}_i<0.05\hfill \end{array}\right. $$

The estimates of the homozygosity intensity ranged from 0 to 1. A higher value indicates a higher homozygosity. Heritability of homozygosity intensity in a region of HD was calculated by using a variance component linkage analysis in MERLIN.

Generalized estimation equation (GEE) analysis was used to examine 855 related individuals and their WGS data, blood pressure (SBP and DBP), HTN, and covariates (age and sex). In the GEE analysis, DBP values, SBP values, and HTN status were modeled as 3 separate response variables. Subsequently, within each sliding window, the association between each response variable and homozygosity intensity was examined separately, with concomitant adjustment for age and sex. An identity link was used in the analysis of SBP and DBP and a logit link was used in the analysis of HTN. Furthermore, we identified gene expression regulated by the significant region(s) of HD. In GEE analysis, each gene expression was modeled as a response variable and its association with homozygosity intensity in the DBP-, SBP-, and HTN-associated regions of HD was examined individually, with concomitant adjustment for age and sex.

We performed simulation studies to evaluate the type 1 error and power of homozygosity association tests. The type 1 error was analyzed by examining the association between Q_1_ and homozygosity intensities, with covariate adjustments for age and sex, by using GEE analysis. The test power was evaluated by examining the association between Q_1_ and homozygosity intensities in the 3 Q_1_-associated regions of HD, with covariate adjustments for age and sex, by using GEE analysis. Throughout the study, the nominal significance level of statistical tests was 0.05 after adjusting for the false discovery rate (FDR) for the multiple-testing problem.

## Results

**Fig. 1:**
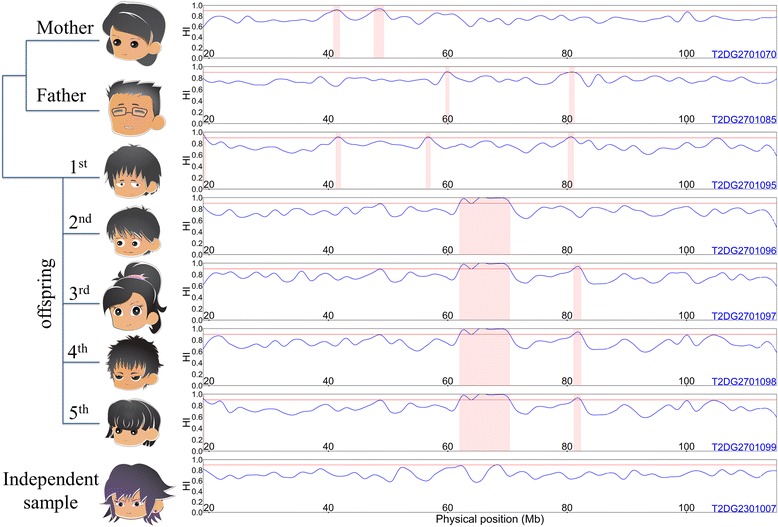
The profiling of homozygosity intensity and regions of HD on chromosome 13. The homozygosity intensity curves of seven individuals in pedigree 27 and 1 independent individual are shown. In each subfigure, the vertical axis represents homozygosity intensity (HI), which ranged from 0 to 1, and the horizontal axis represents the physical positions (Mb) of anchor SNVs of sliding windows on chromosome 13. Blue curves represent homozygosity intensity curves, and the regions shaded in pink correspond to regions of HD

Our analyses were performed without knowledge of the underlying simulation model. We estimated the whole-genome homozygosity intensities of 959 individuals. For example, the profiles of homozygosity intensity of 8 individuals on chromosome 13 are shown (Fig. 1). In summary, 25.86 % of individuals (248/959) carried at least 1 sizable regions of HD, satisfying homozygosity intensity of 0.9 or greater and run length of 5 Mb or greater. The minimum, first quartile, second quartile, third quartile, and maximum of lengths of regions of HD were 5.01 Mb, 5.61 Mb, 6.34 Mb, 7.02 Mb, and 45.78 Mb, respectively. We also calculated the total length of regions of HD carried by an individual and derived the distribution of the total lengths over all individuals. The minimum, first quartile, second quartile, third quartile, and maximum of the total lengths were 5.03, 5.62, 6.42, 7.20, and 108.46 Mb, respectively. We found that the regions of HD and their lengths varied among individuals, but we also observed a pattern of familial aggregation. For example, for the 4 children of pedigree 27, the region of HD between 61.99 and 70.37 Mb on chromosome 13 overlapped (Fig. 1). Average heritability of homozygosity intensity in the region was 86.96 %.

**Fig. 2:**
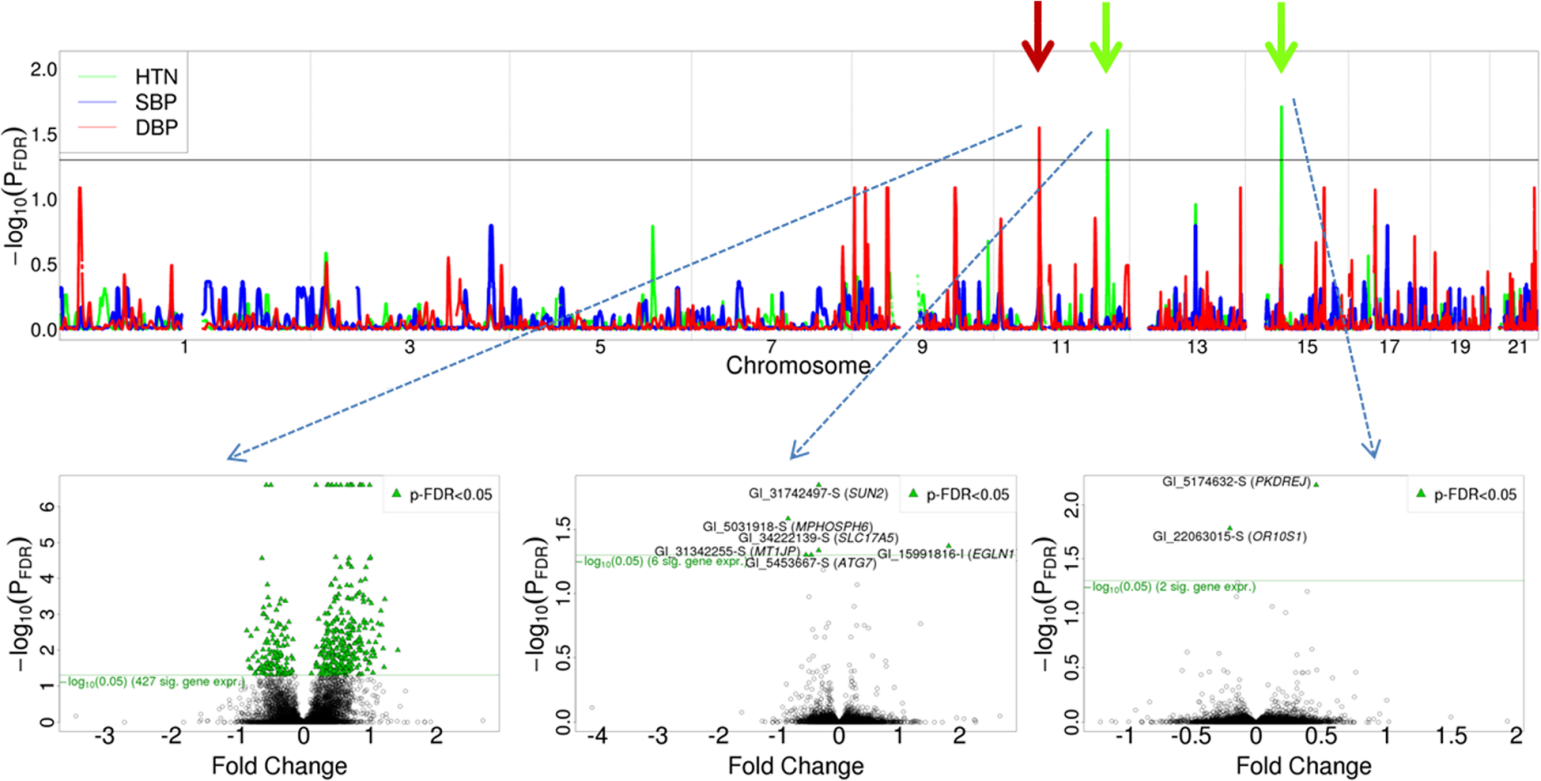
Genetic association between 3 phenotypes and homozygosity intensities, and gene expression regulation of DBP- and HTN-associated regions of HD on odd-numbered chromosomes. The results of homozygosity association tests for DBP (red line), SBP (blue line), and HTN (green line) are shown. The vertical axis represents the *p* values (−log_10_ scale) of the homozygosity association tests after controlling for FDR. The horizontal axis represents the physical positions of the anchor SNVs of sliding windows by chromosome. The *p* values greater than the black reference line reflect statistical significance. Expression regulated by 1 DBP-associated and 2 HTN-associated regions of HD is shown in the 3 volcano plots, respectively. In each volcano plot, the vertical axis represents the *p* values (−log_10_ scale) of generalized estimating equation analysis after controlling for FDR and the horizontal axis represents the fold change of gene expression. Genes with *p* values greater than the green reference line reflect statistical significance and are indicated by green triangles

We performed whole-genome homozygosity association tests to identify DBP-, SBP-, and HTN-associated regions of HD. To examine the multiplicative effect of homozygosity intensity and reduce the influence of outliers, SBP and DBP values were transformed through log transformation and winsorization with a threshold of 0.01. After FDR correction, several DBP- or HTN-associated regions of HD were identified through GEE analysis (Fig. 2). DBP-associated region of HD was located between 45.01 and 45.53 Mb on chromosome 11 (adjusted *p* value = 0.0282). HTN-associated regions of HD were located between 112.60 and 113.43 Mb on chromosome 11 (adjusted *p* value = 0.0295) and between 35.43 and 36.13 Mb on chromosome 15 (adjusted *p* value = 0.019). No SBP-associated regions of HD were identified.

We performed GEE analysis to identify gene expression regulated by the DBP- and HTN-associated regions of HD, with concomitant adjustment for age and sex. The results revealed that the expression of 427 genes was regulated by the DBP-associated region on chromosome 11 (see Fig. 2), where 32 genes were found to be associated with cardiovascular diseases according to the Genetic Association Database (http://geneticassociationdb.nih.gov/). Moreover, the expression of 6 and 2 genes was regulated by the HTN-associated regions on chromosomes 11 and 15, respectively (see Fig. 2).

We evaluated the type 1 error of the homozygosity association tests by analyzing 200 simulation data sets of quantitative trait Q_1_ provided by GAW19. After FDR correction, a homozygosity association analysis of the 849 related individuals calculated the mean of the type 1 errors as 0.0018. We also evaluated the test power by analyzing 3 Q_1_-associated regions of HD in 200 simulation data sets [9]. The results showed that the power increased as the probability *p* increased, where the probability *p* increased as *b*_*1*_ increased. The power of the homozygosity association test was 1.000 in each condition, except for the following 3 parameter combinations (*b*_*0*_, *b*_*1*_) = (−25, 0.4), (−20, 0.3), and (−25, 0.3). The power for the 3 Q_1_-associated regions of HD that represented the 10th, 50th, and 90th percentiles of proportions of RVs on chromosome 21 was: 0.920, 0.985, and 0.775 for (*b*_*0*_, *b*_*1*_) = (−25, 0.4), respectively; 0.630, 0.820, 0.43 for (*b*_*0*_, *b*_*1*_) = (−20, 0.3), respectively; and 0.075, 0.070, and 0.125 for (*b*_*0*_, *b*_*1*_) = (−25, 0.3), respectively. The power for (*b*_*0*_, *b*_*1*_) = (−25, 0.3) was very low because the probability *p* was lower than 0.03 for every individual.

## Discussion

Our previous study examined the genomic patterns of HD in 270 samples from populations with African, Asian, and European ancestry in the International HapMap II Project [7]. The results showed that HD changed significantly among ethnic groups. In the classification analysis, samples from different populations were classified according to the HD results. In contrast, the HD pattern of the African population was least similar to the Asian and white populations; the African population had the fewest regions of HD. In this study, we derived the distribution of HD in the human genome, provided evidence for the genetic component of natural variation in HD, identified regions of HD associated with DBP and HTN, and also identified a set of gene expressions regulated by the DBP- and HTN-associated regions of HD. The follow-up analyses will include, but are not limited to, a copy number analysis and natural selection analysis that will facilitate verifying the genetic mechanism of HD.

## Conclusions

The contributions of this study are summarized as follows: First, we developed a new method for considering the local property and genetic information of homozygosity in homozygosity intensity estimation. In this method, the assumption that RVs have equal importance when defining ROH is relaxed and, the method has a higher power than our previous homozygosity association test [9]. Moreover, this method does not require imputing the homozygosity intensity for the common homozygote of an RV. Therefore, this method is more effective computationally than the previous estimation procedure [9]. Second, our homozygosity intensity analyses of WGS data provide a whole-genome blueprint of HD and evidence for the genetic component of natural variation in HD. Third, the regions and genes of HD associated with DBP and HTN were identified using genome-wide homozygosity association tests, enriching the collection of genes associated with susceptibility to HTN and other blood pressure–related problems. Finally, the association analysis of WGS and WGT data revealed previously unreported evidence of gene regulation of HD. These findings provide a valuable analysis tool and knowledge for future medical genomics and population genomics studies.
